# Metacognition and Mathematical Modeling Skills: The Mediating Roles of Computational Thinking in High School Students

**DOI:** 10.3390/jintelligence12060055

**Published:** 2024-05-31

**Authors:** Jing Zhang, Yu Zhou, Bin Jing, Zhongling Pi, Hongliang Ma

**Affiliations:** 1 Faculty of Education, Shaanxi Normal University, Xi’an 710119, China; zhangjing123@snnu.edu.cn (J.Z.); jingbin@snnu.edu.cn (B.J.); 2 School of Mathematics and Data Science, Changji University, Changji 831100, China; 3 Key Laboratory of Modern Teaching Technology (Ministry of Education), Shaanxi Normal University, Xi’an 710119, China

**Keywords:** metacognitive knowledge, metacognitive experience, metacognitive monitoring, mathematical modeling skills, computational thinking

## Abstract

This study was to investigate the relationship between metacognition and the mathematical modeling skills of high school students, as well as the mediating role of computational thinking. A cluster sampling method was adopted to investigate 661 high school students, using the metacognition scale, computational thinking scale, and mathematical modeling skill test questions. The results showed that metacognitive knowledge and metacognitive monitoring had a direct and positive correlation with high school students’ mathematical modeling skills. Additionally, the critical thinking dimension of computational thinking mediated the relationship between metacognitive knowledge, experience, monitoring, and mathematical modeling skills. These findings indicated that sufficient metacognition could improve the critical thinking of high school students’ computational thinking and enhance their mathematical modeling skills.

## 1. Introduction

In order to improve students’ mathematical literacy, researchers are increasingly focusing on students’ mathematical modeling skills (Anhalt et al. 2018; Schukajlow et al. 2018). Mathematical modeling skills are defined as the capacity to comprehensively apply mathematical knowledge and methods to transform real-world problems into mathematical models for solution and validation (Blum and Ferri 2009; Blomhøj and Jensen 2003; Kaiser et al. 2006). It involves a deep understanding of problems, application of mathematical language, and establishment of models and their solutions (Blum and Leiss 2007; Haines and Crouch 2001; Kaiser 2007). Mathematical modeling skills are closely related to mathematical competency, such as operational proficiency, abstract thinking, logical reasoning, and intuitive imagination (Blum and Leiss 2007; Maaß 2006; Kaiser et al. 2006), and to some extent represents an individual’s mathematical literacy acquired through mathematics education (Geiger et al. 2022; English 2016). It plays a significant role in preparing students to be competitive in the future.

Many scholars have emphasized the importance of metacognition in affecting mathematical modeling skills (Galbraith 2019; Kaiser and Stender 2013; Stillman 2011). Some researchers have pointed out that that metacognition should be considered as part of mathematical modeling skills (Biccard and Wessels 2011). Studies have also shown that students’ mathematical modeling skills are affected by many factors including creativity, cooperativity, algorithmic thinking, problem-solving, and critical thinking (Frejd and Ärlebäck 2011; Lu and Kaiser 2022; Schukajlow et al. 2018). These factors not only play a key role in mathematical modeling but also are considered an important part of computational thinking (Barr and Stephenson 2011; Grover and Pea 2013; Korkmaz et al. 2017; Shute et al. 2017). To our knowledge, the effects of metacognition and computational thinking on the mathematical modeling skills of students have not been tested yet. Only few studies have documented the relationship between these variables and mathematical modeling skills. In the current study, we expand on the previous discussion of mathematical modeling skills by examining the relationship between metacognition and mathematical modeling skills, and the mediating role of computational thinking and its sub-dimensions.

## 2. Literature Review

### 2.1. Metacognition and Mathematical Modeling Skills

Metacognition was introduced in the 1970s by John Flavell and Ann Brown (Flavell 1976, 1979; Brown 1987). According to Flavell’s (1976) model, metacognition is indicated by four major aspects, namely, metacognitive knowledge, experiences, goals, and actions (or approaches). These four dimensions interact with each other. Unlike Flavell’s classification, Brown (1987) categorized metacognition into two parts: metacognitive knowledge and metacognitive control. Based on Flavell and Brown’s classification of metacognition, some researchers have conducted further research on mathematical metacognition (Wang et al. 2017; Wilson and Clarke 2004). For example, Wang et al. (2017) proposed that mathematical metacognition comprises mathematical metacognitive knowledge, experiences, and monitoring, and developed its assessment scales. Regarding mathematical metacognitive knowledge, it involves an individual’s perception of the elements of mathematical learning activities, processes, and outcomes, as well as related knowledge. It typically includes personal knowledge, cognitive tasks, and cognitive strategies (Cui et al. 2018; Wang et al. 2017). Mathematical metacognitive experience is emotional or cognitive experiences that arise during cognitive activities (Wang et al. 2017; Efklides and Petkaki 2005), serving as internal motivators that facilitate the smooth progression of mathematical cognitive activities (Schoenfeld 1987; Tay et al. 2023). Mathematical metacognitive monitoring is at the core of students’ mathematical thinking activities, primarily encompassing four strategic behaviors: planning, regulation, evaluation, and reflection (Wang et al. 2017; Cui et al. 2018).

According to metacognition theory, individuals in the process of cognitive development pay attention to self-awareness and the self-regulation of their cognitive activities (Flavell 1979). Metacognition can assist them in better managing their learning and behavior, leading to improved learning outcomes. Research has shown that metacognition can influence students’ mathematical performance (Kramarski and Mevarech 2003; Mevarech and Fridkin 2006; Schneider and Artelt 2010). Meanwhile, metacognition serves as a bridge between learning content and cognition, facilitating students’ successful resolution of mathematical problems (Özcan 2016; Schoenfeld 2016; Wang et al. 2022). It enables students to effectively plan steps, select strategies, and address problems. Therefore, metacognition has the potential to enhance students’ mathematical modeling skills (Galbraith 2019; Vorhölter 2019; Wendt et al. 2020). According to self-regulated learning theory (Zimmerman 2008), learners can select appropriate strategies and monitor and adjust their learning processes according to goals to achieve optimal outcomes. When students engage in mathematical modeling activities, they need to apply metacognitive strategies to plan their modeling steps, monitor thinking processes, evaluate model rationality, and adjust strategies as needed (Krüger et al. 2020; Stillman 2004). Research has shown that awareness, cognitive strategy, planning, and self-checking of metacognition could facilitate individuals to reflect upon and enhance their mathematical modeling process, identifying issues within it and taking corresponding measures for optimization (Hidayat and Ying 2023; Hidayat et al. 2018). In conclusion, according to metacognition theory (Flavell 1979) and self-regulated learning theory (Zimmerman 2008), we have inferred that metacognition (metacognitive knowledge, metacognitive experience, and metacognitive monitoring) may influence mathematical modeling skills.

### 2.2. Metacognition and Computational Thinking

Metacognition plays a crucial role in learning and problem-solving, as it involves understanding one’s own cognitive processes and the ability to control and monitor these processes effectively (Mayer 1998). Computational thinking refers to a cognitive activity that uses the basic concepts and methods of computer science to solve problems, design systems, and understand human behavior. It emphasizes the use of logical reasoning, algorithm design, and data analysis skills to solve various problems, and is a comprehensive way of thinking (Weintrop et al. 2016; Wing 2006, 2010). Computational thinking is defined as a collective reflection of creativity, algorithmic thinking, critical thinking, problem-solving, cooperativity, and communication skills (ISTE 2015; Korkmaz et al. 2017; Yadav et al. 2022). Creativity refers to the ability of individuals to examine problems from multiple perspectives and use unique ways or strategies to carry out thinking activities (Lu and Kaiser 2022; Aksoy 2004). Algorithmic thinking emphasizes the decomposition, abstraction, and transforming of problems, while optimizing the problem-solving steps to achieve the desired results (Brown 2015; Pala and Mıhçı Türker 2021). Problem-solving is the realization of the planned solution and transference of the problem solution to other situations or domains to solve similar problems efficiently (Grover and Pea 2018; Schoenfeld 2016). Critical thinking is one of the most important cognitive traits for achieving personal success in the 21st century (Saiz and Rivas 2023; Samsudin and Hardini 2019). Cooperativity is a key to solving complex problems, and it enable individuals to effectively communicate, collaborate, and share knowledge with others to collectively advance problem-solving (Møgelvang and Jorun 2023; Molenaar et al. 2014). 

Some researchers have linked creativity to metacognition (Hargrove and Nietfeld 2015; Huang et al. 2021). It is believed that a person with high metacognitive ability would be a more creative problem-solver (Jiang et al. 2023; Mevarech and Paz-Baruch 2022). When teaching programming, metacognition helps students better regulate the process of problem-solving, so as to promote the development of computational thinking (Ma et al. 2021; Pala and Mıhçı Türker 2021; Rum and Ismail 2017). Metacognition influences cooperativity by enhancing students’ self-monitoring and self-regulation abilities, as well as fostering effective teamwork and academic achievement (De Backer et al. 2022; Dindar et al. 2020). There is a strong positive correlation between metacognition and critical thinking (Çakici 2018), which helps learners adjust their plans and strategies during the thinking process and stimulate the development of critical thinking (Magno 2010; Marin and Halpern 2011). According to metacognition theory (Flavell 1976, 1979), individuals enhance their problem-solving abilities by reflecting on their cognitive processes. This reflection helps learners better understand their thinking patterns and behaviors, thus helping to find ways to solve problems (Schraw 1998). Metacognition is an essential factor in the problem-solving process and actively influences problem-solving abilities (Teong 2003). Metacognitive knowledge, skills, and beliefs influence an individual’s own thinking and cognitive processes, thereby improving students’ learning and problem-solving abilities (Livingston 2003; Mayer 1998). Moreover, Joo and Park (2023) confirmed that metacognitive monitoring is a crucial variable influencing computational thinking by analyzing the impact of metacognitive components on computational thinking. Thus, we have inferred that metacognition may influence high school students’ computational thinking.

### 2.3. Computational Thinking and Mathematical Modeling Skills

As previously indicated, computational thinking is regarded as a collective reflection of creativity, algorithmic thinking, cooperativity, critical thinking, and problem-solving abilities (ISTE 2015; Korkmaz et al. 2017). Positioned as a distinctive cognitive approach, computational thinking is frequently intertwined with problem-solving, playing a pivotal role in the mathematical modeling process (Ang 2021). Research has shown a close relationship between creativity and mathematical modeling skills, indicating their mutual influence when solving diverse problems (Lu and Kaiser 2022; Wessels 2014). Algorithmic thinking aids mathematical modelers in comprehending the essence of problems and their key factors, facilitating the selection of suitable algorithms and models for addressing real-world challenges (Barr and Stephenson 2011). Cooperative learning is one of the important factors affecting mathematical problem-solving and mathematical modeling skills (Møgelvang and Jorun 2023). Furthermore, research has shown that critical thinking can help modelers evaluate and validate assumptions, iteratively verify and revise hypotheses and reasoning, and promptly identify and correct errors, thereby enhancing the accuracy and reliability of mathematical modeling (Jablonka 2020). Notably, research confirmed a notable positive correlation between mathematical modeling skills and general problem-solving skills (Koedinger and Anderson 2003). Therefore, we suggest that computational thinking may positively affect mathematical modeling skills.

### 2.4. The Present Study

Based on previous research, the primary goal of this study was to investigate how metacognition factors affect high school students’ mathematical modeling skills. Specifically, this includes two main aims. The first aim is to examine the relationship between metacognition (metacognitive knowledge, metacognitive experience, and metacognitive monitoring) and mathematical modeling skills. The second aim is to explore the mediating role of computational thinking in the relationship between high school students’ metacognition and mathematical modeling skills. The following hypotheses were formulated:

**Hypothesis** **1.** *Metacognitive knowledge is directly and positively related to high school students’ mathematical modeling skills;*

**Hypothesis** **2.** *Metacognitive monitoring is directly and positively related to high school students’ mathematical modeling skills;*

**Hypothesis** **3.** *Metacognitive experience is directly and positively related to high school students’ mathematical modeling skills;*

**Hypothesis** **4.** *Computational thinking exerts a mediating effect on the relationship between metacognition and mathematical modeling skills.*

We focused mainly on the relationship between metacognition and mathematical modeling skills to explore the role of computational thinking as a potential mediator. It was also recognized that the causal analysis of this relationship needs the support of longitudinal data. While the current study provides initial insights, future research will adopt longitudinal designs to further validate and explain the causal relationships between these variables.

## 3. Methods

### 3.1. Sample

This study adopted the cluster sampling method to examine the hypotheses proposed above. First, given the vast territory of western China and the wide distribution of schools, school-based cluster sampling reduces the cost and time for investigators to move between multiple locations compared to simple random sampling. Second, cluster sampling can sample the whole school as a unit based on geographical area, which simplifies the sampling process and improves the research efficiency (Lohr 2021; Young 1986). In this study, an integrated questionnaire, including mathematical modeling skill test, the computational thinking scale, and the mathematical metacognition scale, were distributed among 11th-grade and 12th-grade students in two high schools, and 661 responses were received in total. After filtering out unqualified responses, 572 valid samples remained, resulting in a validity rate of 86.54%. Among these valid samples, 269 (47.02%) of the participants identified as boys, and 303 (52.97%) as girls. A total of 213 (37.24%) students were 11th-grade students, and 359 (70.11%) were 12th-grade students. The mean age of the participants was 17.05 ± 0.65.

### 3.2. Measurements

#### 3.2.1. Mathematical Modeling Skill Test

Frejd (2013) conducted a systematic review and identified that there are several modes of assessment of mathematical modeling skills, including written tests, projects, hands-on tests, portfolios, and contests. Among these, written tests are the most popular, and this approach requires participants to complete a series of modeling questions in a relatively short period of time and assesses their level of mathematical modeling skill. Hidayat et al. (2022) reviewed the literature on the assessment of mathematical modeling education published between 2017 and 2021, and found that the mathematical modeling skill test is still the most commonly used approach to assess students’ mathematical modeling skills. Therefore, we selected high school mathematical modeling test questions to assess modeling skills. Unlike the questions designed for selecting top math students to participate in math modeling competitions, the questions involve basic mathematical knowledge, mathematical thinking methods, and mathematical activity experience. The questions include three items: the “Shoe Size” problem (selected from the Chinese high school Mathematics Curriculum Standards in 2017), the “revolving door problem” (adapted from PISA 2012), and the “Refueling Problem” task (adapted from a task published by Blum and Leiss 2005)—refer to Appendix A for details. Each question is worth 5 points, totaling 15 points. The Cronbach’s *α* coefficient for the test is 0.66, close to 0.67, indicating an acceptable level of internal consistency (Taber 2018).

#### 3.2.2. Computational Thinking Scale

Korkmaz et al. (2017) developed a 22-item scale to assess elementary and middle school students’ self-perception of computational thinking across five dimensions: creativity (4 items), algorithmic thinking (4 items), cooperativity (4 items), critical thinking (4 items), and problem-solving (4 items), based on the definition of computational thinking by the ISTE (2015). Responses were rated on a 5-point scale: 1 for strongly disagree and 5 for strongly agree. The Cronbach’s *α* coefficients for the overall scale and each dimension were 0.86, 0.65, 0.74, 0.86, 0.79, and 0.81, respectively, which mostly aligned with the acceptable range of 0.67 to 0.87 as stated by Taber (2018), indicating adequate internal consistency.

#### 3.2.3. Metacognition Scale

Metacognition was measured using the mathematical metacognition scale (Wang et al. 2017). The questionnaire contained 50 items divided into three dimensions: metacognitive knowledge (14 items in total, including three sub-dimensions, namely, knowledge about individuals, knowledge about tasks, and knowledge about strategies), metacognitive experience (9 items in total, including two sub-dimensions, namely, cognitive experience and affective experience), and metacognitive monitoring (27 items in total, including five sub-dimensions, namely, planning, regulation, evaluation, inspection, and management). Each item was ranked on a 5-point Likert scale from 1 = strongly disagree to 5 = strongly agree. The mean score was calculated for each participant, with higher scores indicating a high level of metacognition. The overall Cronbach’s *α* of the questionnaire was 0.98, of the metacognitive knowledge subscale was 0.95, of the mathematical metacognitive experience subscale was 0.93, and of the mathematical metacognitive monitoring subscale was 0.97.

### 3.3. Data Collection and Analysis

Prior to data collection, all participants were invited to complete an informed consent form. All the data of the participants were collected through paper and pencil tests. After data collection was completed, we used SPSS 27.0 and Mplus 8.3 statistical tools to analyze the data. Specifically, descriptive statistical analysis and correlation analysis were first implemented. Then, a confirmatory factor analysis (*CFA*) was conducted to assess the reliability and validity of the measurement model. Finally, the fit indicators of the structural model in Mplus were assessed using structural equation modeling (*SEM*). Considering the effect of gender and grade on mathematical modeling skills, we included gender and grade as two control variables in the model. Regarding *CFA* and *SEM*, chi-square (*χ*^2^), degree of freedom (*df*), *χ*^2^*/df*, root mean square error of approximation (*RMSEA*), the comparative fit index (*CFI*), the Tucker Lewis index (*TLI*), and standardized root mean residual (*SRMR*) are commonly used as the fitting indicators that must be reported in general research. The cutoff values recommended for the model fit metrics were: *χ*^2^*/df* ≤ 5 (more stringent standards require ≤ 3), *CFI* ≥ 0.90, *TLI* ≥ 0.90, *RMSEA* ≤ 0.06, and *SRMR* ≤ 0.10 (Meyers et al. 2016).

## 4. Results

### 4.1. Assessment of Measurement Model

We used item reliability, the model fit index, convergent validity, composite reliability (*CR*), and discriminant validity to assess the measurement model. Among them, item reliability and convergent validity were measured using the square of item factor loading (*R*^2^) and average variance extracted (*AVE*), respectively. The value of *AVE* is determined through the formula $AVE=\frac{(\sum \lambda^{2})}{N}$, where λ = factor loading and *N* = number of indicators to be measured. The value of *CR* is determined through the formula $CR=\frac{{\left(\sum \lambda \right)}^{2}}{{\left(\sum \lambda \right)}^{2}+\sum \varepsilon }$, where *λ* = factor loading and *ε* = measurement error.

As shown in Table 1, all factor loadings were greater than 0.60 and the *p*-values were statistically significant. The *R*^2^ of most items exceeded 0.50 or was close to 0.50, meaning that all items were acceptable. The fitting index of most scales also reached the recommended threshold, which can also be considered a good fit. The *CR* values of the variables ranged from 0.76 to 0.96, which all exceeded the recommended cutoff value of 0.60 (Bagozzi and Yi 1988), indicating that the items of the constructs had good internal consistency. Most of the *AVE* values were greater than 0.50, meeting the recommended criterion of 0.50 (Fornell and Larcker 1981). Although the *AVE* value of the mathematical modeling skill (*MMS*) construct was slightly less than 0.50, the *CR* value exceeded 0.70, which indicated that the convergence validity was acceptable (Lam 2012).

As shown in Table 2, there was a significant positive correlation between the three factors of metacognition (metacognitive knowledge, metacognitive experience, and metacognitive monitoring) and mathematical modeling skills (*p* < 0.001). The factors of creativity, algorithmic thinking, cooperativity, critical thinking, and problem-solving in computational thinking were positively correlated with mathematical modeling skills (*p* < 0.001). The three factors of mathematical metacognition were significantly positively correlated with the five factors of computational thinking (*p* < 0.001). The results of correlation test support the follow-up analysis of *SEM*.

In addition, due to the high correlation between the variables, the discriminant validity was also assessed in this study. According to the criterion proposed by Fornell and Larcker (1981), if the square roots of the *AVE* of each dimension (variable) are greater than the “maximum value of correlation coefficient of the dimension with other dimensions”, then it indicates good discriminant validity. As shown in Table 2 below, the square roots of the *AVE* of variables were mostly greater than the correlation coefficients of the related variables, indicating that the discriminant validity of the measurement model in this study is adequate.

### 4.2. Assessment of Structural Model

In this study, a direct effect hypothesis model and a mediation effect hypothesis model were constructed. In addition, we compared the two hypothesis models with their corresponding one-factor models, respectively. For comparisons between one-factor models and hypothesis models, two aspects are usually considered. One is that the fit index becomes better, and the other is that the dependent variable is more explained. By comparing the fit index, Akaike information criterion (*AIC*), and *R*^2^ of the one-factor model and the hypothesis model, we determined the suitability. According to Table 3, the fit indexes and *AIC* values of the two one-factor models are almost greater than those of the two hypothesis models, respectively. Also, the explanation rate *R*^2^ of the two one-factor models is relatively low. It is implied that the direct effect hypothesis model and the mediation effect hypothesis model constructed in this study are more suitable.

**Figure 1:**
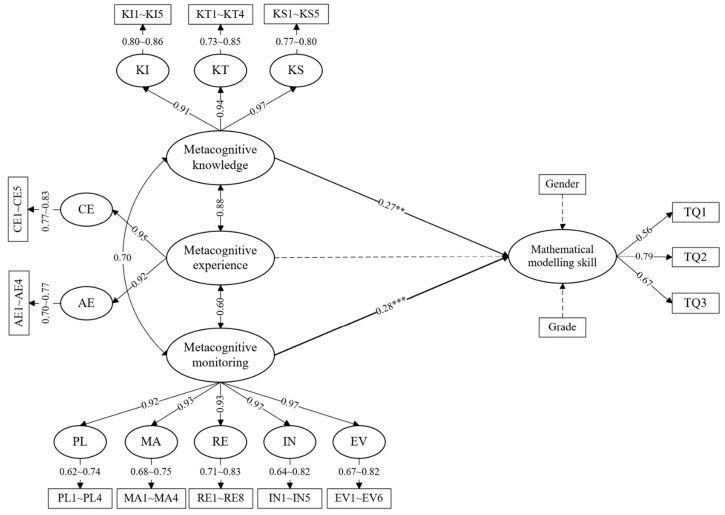
Direct hypothesis model of metacognitive knowledge, metacognitive experience, and metacognitive monitoring on mathematical modeling skills. (Note: The coefficients shown are standardized path coefficients. A solid arrow represents a significant path, and a dashed arrow represents a nonsignificant path. All nonsignificant path coefficients were deleted to enhance the conciseness of the model. ** *p* < 0.01 and *** *p* < 0.001).

Figure 1 presents the results of hypothesis testing for the path coefficients of the direct effect hypothesis model. The results indicated that metacognitive knowledge (*β* = 0.27, *p* < 0.01) and metacognitive monitoring (*β* = 0.28, *p* < 0.001) significantly and positively affected mathematical modeling skills; the results supported hypotheses 1 and 2. However, metacognitive experience did not influence mathematical modeling skills; this result did not support hypothesis 3.

**Figure 2:**
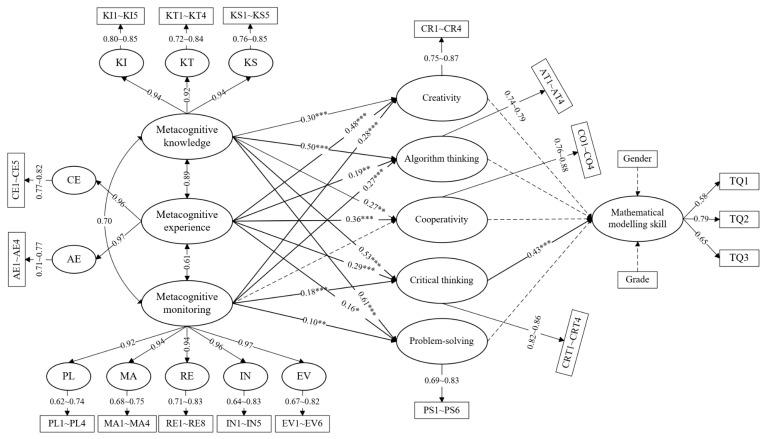
Mediation hypothesis model of metacognitive knowledge, metacognitive experience, and metacognitive monitoring on mathematical modeling skills. (Note: * *p* < 0.05, ** *p* < 0.01 and *** *p* < 0.001).

The mediation effects of the five sub-dimensions of computational thinking on metacognitive knowledge, metacognitive experience, and metacognitive monitoring were also examined. As shown in Figure 2, the results demonstrated that metacognition (including metacognitive knowledge, metacognitive monitoring, and metacognitive experience) had a positive effect on critical thinking (0.18 < *β* < 0.53 and *p* < 0.001), and critical thinking had a positive effect on mathematical modeling skills (*β* = 0.43 and *p* < 0.001). This means that the effect of metacognition on mathematical modeling skills was mediated by critical thinking. However, the other sub-dimensions of computational thinking, including creativity, algorithmic thinking, cooperativity, and problem-solving, had no obvious mediating effects on the relationship between metacognition and mathematical modeling skills. These results partially support hypothesis 4.

### 4.3. Mediating Effect Analysis

As shown in Figure 2, metacognition had a positive effect on critical thinking, which further positively influenced mathematical modeling skills. Therefore, we further tested the mediating effect of critical thinking using a Bootstrap method with 2000 repeated samples and a confidence level of 95% in Mplus software. The results are given in Table 4. As shown in Table 4, the mediating effect value of critical thinking between metacognitive knowledge and mathematical modeling skills was 0.18. The 95% confidence interval was [0.07, 0.33]. The mediating effect value of critical thinking between metacognitive experience and mathematical modeling skills was 0.08, with a 95% confidence interval of [0.02, 0.20], and the mediating effect value of critical thinking between metacognitive monitoring and mathematical modeling skills was 0.05, with a 95% confidence interval of [0.02, 0.10]. Therefore, critical thinking had a significant mediating effect between metacognition and mathematical modeling skills. This further validated that hypothesis 4 was partially supported.

## 5. Discussion

This study explored the mediating effect of computational thinking on the influence between metacognition and mathematical modeling skills through *SEM*. The results showed that metacognition could influence critical thinking and thus mathematical modeling skills. These results means that we may improve students’ metacognition, and thus their mathematical modeling skills, through critical thinking. These findings may provide a useful reference for teachers to train students’ mathematical modeling skills. 

Firstly, we found that metacognitive knowledge in mathematics significantly and positively influenced high school students’ mathematical modeling skills, which is consistent with previous findings (Stillman 2004, 2011; Stillman and Galbraith 1998; Veenman 2006). Stillman (2011) explored the key elements of metacognitive knowledge that play a significant role in the mathematical modeling process. The individual element emphasizes the difficulty of being aware of reasonable estimates when modeling, while the task element focusses on identifying the specific features that affect task solutions. In addition, the strategy element refers to the modeler’s evaluation and selection of the effectiveness of the strategy based on experience. This suggests that metacognitive knowledge is crucial to mathematical modeling skills, which not only helps modelers to deeply analyze their own performance in the modeling process, but also guides them to accurately identify task characteristics and to improve the accuracy and efficiency of mathematical modeling. Furthermore, an individual’s metacognitive knowledge develops with age and experience (Veenman and Verheij 2003). In the context of traditional Chinese mathematics education, which emphasizes fundamental knowledge and basic skills (Li et al. 2019; Cheng et al. 2021), students at the high school level have already accumulated a certain amount of metacognitive knowledge in mathematics. This knowledge could deepen individuals’ understanding of mathematical modeling, thereby exerting a positive influence on mathematical modeling skills. 

In previous studies, metacognitive monitoring has been considered a core compo-nent of metacognition (Wang et al. 2017), capable of influencing students’ mathematical modeling skills (Vorhölter 2018; Schukajlow et al. 2018). This study supports hypothesis 2 and further confirms existing studies. The positive effect on metacognitive monitoring and mathematical modeling skills may be due to the fact that metacognitive monitoring can facilitate strategy use (Künsting et al. 2013). There exists a discernible correlation between the adoption of these strategies and the corresponding task performance (Luwel et al. 2003). Metacognitive monitoring is effective in improving mathematical modeling skills by moderating student’s understanding of the task and the successful application experience of the strategy. Thus, metacognitive monitoring may influence mathematical modeling skills by moderating students’ understanding of the task and experience of successful application of strategies. Krüger et al. (2020) argue that applying metacognitive strategies during the conceptualization phase of the mathematical modeling process can provide students with a sense of guidance. Educators may incorporate metacognitive monitoring strategies into their teaching practices to help students develop their mathematical modeling skills to a higher level.

Regarding metacognitive experience, previous research has indicated a close correlation between metacognitive experience and mathematical problem-solving (Scheibe et al. 2023). Metacognitive experience plays a crucial role in students’ cognition, emotions, self-concept, and problem-solving abilities (Akama and Yamauchi 2004; Efklides et al. 1999). Different from these existing research findings, the current study’s results indicate that metacognitive experience has no direct influence on mathematical modeling skills. The primary reason for this disparity may lie in the fact that metacognitive experience is cognitive or emotional experiences accompanying cognitive activities, and their duration varies. They may occur during sustained periods of cognitive activities, or before or after cognitive activities (Efklides and Petkaki 2005; Flavell 1979). These experiences may change according to the difficulty level of the tasks as perceived by students. However, in the current study, metacognitive experience is only measured prior to addressing mathematical modeling problems, and this may not fully capture the potential impact of metacognitive experience on mathematical modeling skills.

Mediation analysis revealed that metacognitive knowledge, as well as metacognitive monitoring and metacognitive experience, indirectly impacts high school students’ mathematical modeling skills through the critical thinking component of computational thinking. This indicates that the higher the level of metacognition and the stronger the critical thinking, the higher the mathematical modeling skills of high school students. Magno (2010) investigated the relationship between metacognition and critical thinking through empirical research and found a significant positive correlation between the two factors. Other relevant studies have also demonstrated a significant positive correlation between metacognition and critical thinking (Ku and Ho 2010; Samsudin and Hardini 2019; Teng and Yue 2023). These findings align with the results of the current study. According to metacognitive theory (Flavell 1976, 1979), an individual’s cognitive activities encompass a series of cognitive processes, emphasizing the individual’s awareness and control of their thinking processes and outcomes (Schraw and Moshman 1995). Therefore, the stronger the metacognitive abilities, the more effectively individuals adjust and optimize their thinking processes with the assistance of metacognitive knowledge, experiences, and monitoring, leading to higher levels of critical thinking. Additionally, according to constructivist learning theory, learning is the process by which learners actively construct and comprehend new knowledge based on their existing knowledge and experiences (Bada and Olusegun 2015). Critical thinking skills, as essential skills for 21st century students (Saiz and Rivas 2023), help students understand the transition between real-life situations and mathematics, and facilitate the design of unique problem-solving approaches. This implies that the stronger the critical thinking, the stronger the mathematical modeling skills (Kannadass et al. 2023). The above findings indicate that the higher the levels of mathematical metacognitive knowledge, metacognitive experience, and metacognitive monitoring among high school students, the stronger their critical thinking, leading to further enhancements of their mathematical modeling skills. 

Our findings reveal the importance of metacognition and computational thinking in developing mathematical modeling skills. First, we confirm the direct positive effect of metacognitive knowledge and monitoring on mathematical modeling skills, which emphasizes the value of strengthening students’ metacognition in mathematical modeling education. Second, this study further reveals the role of critical thinking of computational thinking as a bridge between metacognition and mathematical modeling skills. This means that the improvement of metacognition also has a positive impact on mathematical modeling skills indirectly by promoting critical thinking. Therefore, in the practice of mathematical modeling education, we strongly suggest that teachers pay attention to cultivating students’ metacognition and integrate critical thinking activities into the teaching practice.

## 6. Limitations and Directions for Future Research

There are several limitations in this study that should be acknowledged. First, the sample size of participants was insufficient to generalize the findings to other regions or countries. To ensure the scalability and stability of the conclusions, future studies can be expanded to schools in different regions or countries, leading to broader guidance for a larger range of mathematical educational practices. Second, this study is a cross-sectional study, which can only verify the mediating effect among variables but cannot prove a causal relationship between variables. Recent studies have indicated that metacognition can influence computational thinking and, in turn, that computational thinking is also an influential factor in metacognition (Markandan et al. 2022; Román-González et al. 2017). Therefore, a longitudinal study can be considered in future research to examine the causal relationship between variables based on their bidirectional effects. Third, while this study focused primarily on assessing the differences between students within groups, it did not explore the possible differences between groups of students, such as those based on different grade levels, learning abilities, or other demographic variables. By understanding within- and between-group differences, the understanding of how metacognition and computational thinking influence mathematical modeling skills can be improved. Therefore, conducting multilevel *SEM* analyses would be beneficial to future research. Finally, our approach to assessing the construct validity of the scales has some limitations. We only focused on each item of the variables regarding the convergent validity, while discriminant validity focused on the scale scores of the variables. Even though all scales used in this study were well-developed scales with good reliability and validity that have been verified and have been widely implemented in the Chinese context, the convergent and discriminant validities still need to be determined analytically with the multitrait–multimethod (*MTMM*) approach (Campbell and Fiske 1959; Krabbe 2016, p. 118; Ziegler 2020), which would be instructive for our future research.

## Figures and Tables

**Table 1:** The reliability and convergence validity of the measurement model.

Measurement Model			Significance Test of Parameters				*R* ^2^	Fit Index	*AVE*	*CR*
			Estimate	S.E.	Est./S.E.	*p*				
*MK*	*KI*	*KI1*	0.80	0.01	62.81	***	0.76	*χ*^2^ = 219.34, *df* = 74, χ^2^/df = 2.96, *RMSEA* = 0.06, *CFI* = 0.97, *TLI* = 0.97, *SRMR* = 0.03	0.65	0.96
		*KI2*	0.82	0.01	63.04	***	0.66			
		*KI3*	0.86	0.01	79.90	***	0.77			
		*KI4*	0.80	0.01	56.72	***	0.57			
		*KI5*	0.82	0.01	68.09	***	0.61			
	*KT*	*KT1*	0.84	0.01	70.40	***	0.63			
		*KT2*	0.80	0.01	60.24	***	0.61			
		*KT3*	0.83	0.01	64.68	***	0.54			
		*KT4*	0.73	0.02	45.35	***	0.66			
	*KS*	*KS1*	0.76	0.02	45.94	***	0.68			
		*KS2*	0.77	0.02	50.68	***	0.77			
		*KS3*	0.76	0.02	51.07	***	0.57			
		*KS4*	0.85	0.01	74.73	***	0.65			
		*KS5*	0.80	0.01	58.53	***	0.80			
*ME*	*CE*	*CE1*	0.82	0.01	61.55	***	0.82	*χ*^2^ = 86.61, *df* = 26, *χ*^2^*/df* = 3.33, *RMSEA* = 0.06, *CFI* = 0.98, *TLI* = 0.97, *SRMR* = 0.02	0.66	0.95
		*CE2*	0.82	0.01	69.47	***	0.69			
		*CE3*	0.77	0.01	57.42	***	0.55			
		*CE4*	0.82	0.01	64.58	***	0.61			
		*CE5*	0.77	0.01	57.11	***	0.66			
	*AE*	*AE1*	0.88	0.01	122.87	***	0.64			
		*AE2*	0.85	0.01	81.12	***	0.74			
		*AE3*	0.81	0.01	68.23	***	0.54			
		*AE4*	0.76	0.01	62.93	***	0.64			
*MM*	*PL*	*PL1*	0.62	0.02	28.55	***	0.67	*χ*^2^ = 1070.41, *df* = 317, *χ*^2^*/df* = 3.38, *RMSEA* = 0.06, *CFI* = 0.93, *TLI* = 0.92, *SRMR* = 0.04	0.57	0.97
		*PL2*	0.74	0.02	43.80	***	0.73			
		*PL3*	0.70	0.02	34.91	***	0.64			
		*PL4*	0.70	0.02	35.54	***	0.68			
	*MA*	*MA1*	0.73	0.02	41.63	***	0.71			
		*MA2*	0.68	0.02	35.38	***	0.63			
		*MA3*	0.75	0.02	50.70	***	0.69			
		*MA4*	0.73	0.02	32.54	***	0.53			
	*RE*	*RE1*	0.78	0.01	55.01	***	0.58			
		*RE2*	0.77	0.02	52.32	***	0.60			
		*RE3*	0.76	0.02	50.60	***	0.58			
		*RE4*	0.71	0.02	41.70	***	0.72			
		*RE5*	0.77	0.02	49.23	***	0.64			
		*RE6*	0.83	0.01	66.64	***	0.66			
		*RE7*	0.78	0.02	52.46	***	0.67			
		*RE8*	0.78	0.02	51.33	***	0.59			
	*IN*	*IN1*	0.79	0.01	60.34	***	0.68			
		*IN2*	0.80	0.01	62.91	***	0.59			
		*IN3*	0.64	0.02	29.78	***	0.77			
		*IN4*	0.83	0.01	62.47	***	0.72			
		*IN5*	0.72	0.02	38.67	***	0.65			
	*EV*	*EV1*	0.79	0.01	58.77	***	0.58			
		*EV2*	0.82	0.01	73.44	***	0.38			
		*EV3*	0.67	0.02	33.59	***	0.55			
		*EV4*	0.81	0.01	65.41	***	0.49			
		*EV5*	0.81	0.01	64.26	***	0.49			
		*EV6*	0.80	0.02	54.64	***	0.53			
*CT*	*CR*	*CR1*	0.87	0.01	78.34	***	0.46	*χ*^2^ = 4.04, *df* = 2, *χ*^2^*/df* = 2.01, *RMSEA* = 0.04, *CFI* = 0.99, *TLI* = 0.99, *SRMR* = 0.01	0.69	0.90
		*CR2*	0.81	0.01	77.91	***	0.56			
		*CR3*	0.88	0.01	98.28	***	0.53			
		*CR4*	0.75	0.02	48.69	***	0.60			
	*AT*	*AT1*	0.78	0.02	52.47	***	0.60	*χ*^2^ = 6.26, *df* = 2, *χ*^2^*/df* = 3.13, *RMSEA* = 0.06, *CFI* = 0.99, *TLI* = 0.97, *SRMR* = 0.02	0.60	0.86
		*AT2*	0.79	0.02	52.33	***	0.57			
		*AT3*	0.78	0.01	54.76	***	0.51			
		*AT4*	0.74	0.02	41.84	***	0.60			
	*CO*	*CO1*	0.81	0.01	58.78	***	0.69	*χ*^2^ = 6.37, *df* = 2, *χ*^2^*/df* = 3.19, *RMSEA* = 0.06, *CFI* = 0.99, *TLI* = 0.98, *SRMR* = 0.02	0.67	0.89
		*CO2*	0.83	0.02	54.48	***	0.61			
		*CO3*	0.88	0.01	77.34	***	0.61			
		*CO4*	0.76	0.02	46.92	***	0.63			
	*CRT*	*CRT1*	0.80	0.01	65.01	***	0.64	*χ*^2^ = 6.52, *df* = 2, *χ*^2^*/df* = 3.26, *RMSEA* = 0.06, *CFI* = 0.99, *TLI* = 0.97, *SRMR* = 0.01	0.74	0.92
		*CRT2*	0.89	0.01	91.58	***	0.41			
		*CRT3*	0.91	0.01	115.62	***	0.68			
		*CRT4*	0.83	0.01	71.49	***	0.53			
	*PS*	*PS1*	0.74	0.02	44.97	***	0.63	*χ*^2^ = 13.74, *df* = 7, *χ*^2^*/df* = 1.96, *RMSEA* = 0.04, *CFI* = 0.99, *TLI* = 0.99, *SRMR* = 0.01	0.62	0.91
		*PS2*	0.78	0.02	52.04	***	0.68			
		*PS3*	0.81	0.01	60.88	***	0.45			
		*PS4*	0.80	0.01	56.06	***	0.66			
		*PS5*	0.86	0.01	79.58	***	0.66			
		*PS6*	0.73	0.02	47.14	***	0.65			
*MMS*		*TQ1*	0.58	0.03	21.29	***	0.41	—	0.46	0.72
		*TQ2*	0.79	0.02	36.29	***	0.62			
		*TQ3*	0.65	0.03	25.05	***	0.42			

Note: *MK*—metacognitive knowledge; *KI*—knowledge about individuals; *KT*—knowledge about tasks; *KS*—knowledge about strategies; *ME*—metacognitive experience; *CE*—cognitive experience; *AE*—affective experience; *MM*—metacognitive monitoring; *PL*—planning; *MA*—management; *RE*—regulation; *IN*—inspection; *EV*—evaluation; *CT*—computational thinking; *CR*—creativity; *AT*—algorithmic thinking; *CO*—cooperativity; *CRT*—critical thinking; *PS*—problem-solving; *MMS*—mathematical modeling skill; *TQ*—test question; *** *p* < 0.001.

**Table 2:** Descriptive statistics of each variable, correlation analysis, and discriminant validity of the measurement model.

	M ± *SD*	1	2	3	4	5	6	7	8
1 Metacognitive knowledge	3.45 ± 0.81	**0.81**							
2 Metacognitive experience	3.20 ± 0.88	0.81 ***	**0.81**						
3 Metacognitive monitoring	3.09 ± 0.80	0.64 ***	0.55 ***	**0.75**					
4 Creativity	3.61 ± 0.91	0.82 ***	0.82 ***	0.71 ***	**0.83**				
5 Algorithmic thinking	3.28 ± 0.83	0.74 ***	0.69 ***	0.66 ***	0.75 ***	**0.77**			
6 Cooperativity	3.44 ± 0.82	0.53 ***	0.55 ***	0.38 ***	0.56 ***	0.52 ***	**0.86**		
7 Critical thinking	3.37 ± 1.01	0.80 ***	0.77 ***	0.65 ***	0.78 ***	0.73 ***	0.52 ***	**0.79**	
8 Problem-solving	3.40 ± 0.83	0.72 ***	0.68 ***	0.56 ***	0.70 ***	0.64 ***	0.52 ***	0.72 ***	**0.68**
9 Mathematical modeling skills	8.45 ± 4.09	0.46 ***	0.41 ***	0.43 ***	0.48 ***	0.43 ***	0.34 ***	0.50 ***	0.40 ***

Note: *M* = Means, *SD* = standard deviations, and *** *p* < 0.001. The bold number is the square roots of the *AVE*.

**Table 3:** Comparison of one-factor and hypothesis model fit index.

Model	*χ* ^2^	*df*	*χ*^2^/*df*	*RMSEA*	*CFI*	*TLI*	*SRMR*	*AIC*	*R* ^2^
One-factor direct effect model	4903.34	1423	3.45	0.07	0.84	0.83	0.07	72,485.36	0.35
Direct effect hypothesis model	4262.65	1413	3.02	0.06	0.87	0.87	0.06	71,823.21	0.37
One-factor mediation effect model	9011.03	2825	3.19	0.06	0.82	0.81	0.07	98,626.99	0.38
Mediation effect hypothesis model	8254.91	2813	2.93	0.06	0.94	0.93	0.06	97,840.42	0.40

**Table 4:** Mediating effects among variables.

Outcome Variable	Predictor Variable	Mediator	Indirect Effect	SE	95% Confidence Level	
					Lower	Upper
Mathematical modeling skills	Metacognitive knowledge	Creativity	0.05	0.04	−0.01	0.14
		Algorithmic thinking	0.04	0.05	−0.06	0.15
		Cooperativity	0.02	0.02	−0.01	0.08
		Critical thinking	0.18	0.07	0.07	0.33
		Problem-solving	0.03	0.05	−0.05	0.12
	Metacognitive experience	Creativity	0.08	0.07	−0.03	0.22
		Algorithmic thinking	0.02	0.03	−0.02	0.08
		Cooperativity	0.03	0.03	−0.01	0.11
		Critical thinking	0.08	0.04	0.02	0.20
		Problem-solving	0.01	0.02	−0.01	0.18
	Metacognitive monitoring	Creativity	0.05	0.04	−0.03	0.05
		Algorithmic thinking	0.02	0.03	−0.01	0.13
		Cooperativity	0.001	0.01	−0.01	0.02
		Critical thinking	0.05	0.02	0.02	0.10
		Problem-solving	0.01	0.01	−0.01	0.04

## Data Availability

The original contributions presented in the study are included in the article, further inquiries can be directed to the corresponding author.

## Appendix A

### Appendix A1. Shoe Size

People often see the following table while buying shoes online. The first line can be interpreted as the length of the foot; the second line is what we are used to calling “shoe size”.

International shoe size (mm)	220	225	230	235	240	245	250	255	260	265
Common use (European size)	34	35	36	37	38	39	40	41	42	43

Please answer the following questions

(1) Find a function model that satisfies the above conditions.
(2) What is the actual length of a foot that wears children’s shoes in customarily called “size 30”?
(3) A basketball player’s foot is 282 mm long. What size shoe should he wear?

### Appendix A2. Revolving Door

A revolving door consists of three glass doors capable of rotating within a cylindrical space. The inside diameter of this space is 2 m (200 cm). The diameter of this space is 2 m (200 cm), and the three glass doors divide the cylindrical space into three equal smaller spaces. Below is a top view of the revolving door in three different positions (below left). The entrance and exit are of the same size (shown as dotted lines in the right diagram below), and if the entrance and exit are too large, this revolving glass door will not provide an enclosed space in which air can circulate freely, resulting in unnecessary heat loss, as shown in the diagram on the right below.

Q: What is the maximum arc length at the entrance that will make it possible for air never to flow freely between the entrance and exit?

### Appendix A3. Fuel Filling

Mr. Wang usually drives a Honda 180TURBO, and there is a gas station A near his home, and a gas station B 10 kilometers away from his home, and the price of No. 95 gasoline at both gas stations A and B this week is RMB 7.60/L. He has previously applied for a free membership of gas station B, and can enjoy a discount of CNY 0.4 less per liter of gasoline. The following chart shows some of the parameters of the Honda 180TURBO. Please answer the following questions: Analyze which gas station should Mr. Wang choose to fill up his car more cost-effectively?
